# Understanding Views Around the Creation of a Consented, Donated Databank of Clinical Free Text to Develop and Train Natural Language Processing Models for Research: Focus Group Interviews With Stakeholders

**DOI:** 10.2196/45534

**Published:** 2023-05-03

**Authors:** Natalie K Fitzpatrick, Richard Dobson, Angus Roberts, Kerina Jones, Anoop D Shah, Goran Nenadic, Elizabeth Ford

**Affiliations:** 1 Institute of Health Informatics University College London London United Kingdom; 2 Department of Biostatistics and Health Informatics King’s College London London United Kingdom; 3 Department of Population Data Science Swansea University Medical School Swansea United Kingdom; 4 University College London Hospitals NHS Foundation Trust London United Kingdom; 5 Department of Computer Science University of Manchester Manchester United Kingdom; 6 Brighton and Sussex Medical School Brighton United Kingdom

**Keywords:** consent, databank, electronic health records, free text, governance, natural language processing, public involvement, unstructured text

## Abstract

**Background:**

Information stored within electronic health records is often recorded as unstructured text. Special computerized natural language processing (NLP) tools are needed to process this text; however, complex governance arrangements make such data in the National Health Service hard to access, and therefore, it is difficult to use for research in improving NLP methods. The creation of a donated databank of clinical free text could provide an important opportunity for researchers to develop NLP methods and tools and may circumvent delays in accessing the data needed to train the models. However, to date, there has been little or no engagement with stakeholders on the acceptability and design considerations of establishing a free-text databank for this purpose.

**Objective:**

This study aimed to ascertain stakeholder views around the creation of a consented, donated databank of clinical free text to help create, train, and evaluate NLP for clinical research and to inform the potential next steps for adopting a partner-led approach to establish a national, funded databank of free text for use by the research community.

**Methods:**

Web-based in-depth focus group interviews were conducted with 4 stakeholder groups (patients and members of the public, clinicians, information governance leads and research ethics members, and NLP researchers).

**Results:**

All stakeholder groups were strongly in favor of the databank and saw great value in creating an environment where NLP tools can be tested and trained to improve their accuracy. Participants highlighted a range of complex issues for consideration as the databank is developed, including communicating the intended purpose, the approach to access and safeguarding the data, who should have access, and how to fund the databank. Participants recommended that a small-scale, gradual approach be adopted to start to gather donations and encouraged further engagement with stakeholders to develop a road map and set of standards for the databank.

**Conclusions:**

These findings provide a clear mandate to begin developing the databank and a framework for stakeholder expectations, which we would aim to meet with the databank delivery.

## Introduction

### Background

Electronic health records (EHRs) contain a rich narrative of the patient journey and have huge potential for research [1]. However, research using EHRs is typically limited to the structured data (such as numerical values and diagnoses coded using a controlled vocabulary), despite a large proportion of the information in EHRs being in the form of unstructured (free) text. The analysis of free text at scale requires specialized tools and methods (natural language processing [NLP]) to “read,” process, and structure the information before it can be used at scale for research purposes.

NLP of clinical text has many potential benefits, both for individual care and improving health services [2]. These include (1) to facilitate the process of clinical coding [3], which is the process by which clinical coding staff in hospitals assign codes from a specific terminology (eg, International Classification of Diseases, 10th revision [ICD-10]) [4] to patient episodes for reimbursement; (2) to facilitate structured recording of diagnoses in clinical care using Systematized Nomenclature of Medicine Clinical Terms (SNOMED CT) [5], which is currently not done consistently [6]; and (3) to enable research using information in EHRs, which are currently not coded. Compared with manual review of free text, automated analysis is much faster and enables a much larger amount of text to be analyzed, enabling larger and more representative patient samples to be used for research.

### The Challenge

Tools and platforms for text access and analysis such as CogStack (developed by a consortia of scientists at King’s College London, King’s College Hospital, South London and the Maudsley, Guy’s and St Thomas’s hospital, University College London, and University College London Hospitals National Health Service [NHS] Foundation Trust and some members of the CogStack open-source community) [7] have been developed and installed at some NHS sites with great success, but overall, access to free text for researchers is still currently difficult. Ideally, free text needs to be brought out of the NHS environment so expert computer scientists working in university research or other non-NHS environments can use the data to train their computer algorithms to extract the important clinical information. In the United Kingdom, the application of NLP for health care text research is largely limited to within large NHS hospital trusts with academic affiliations and in-house NLP expertise owing to complex governance requirements arising from increased concerns around the potential risk of reidentifying patients. In Scotland, a successful model adopted by groups including the Health Informatics Centre at the University of Dundee [8], DataLoch [9], and the national electronic Data Research and Innovation Service [10] in collaboration with the University of Edinburgh Clinical NLP Research Group [11] involves the provision of data for research through secure trusted research environments outside NHS or university settings. Nind et al [12] describe an approach for extracting, linking, deidentifying, and hosting clinical imaging data within a controlled secure environment as a resource for national and international research. This model provides a potential alternative approach to hosting the databank outside of an NHS or university setting and allows for timely and secure access to data; however, the governance framework is complex. In the United Kingdom, before medical data can be shared outside of the NHS environment, identifiers such as names of patients, family members, and health care professionals; addresses; and dates of birth, which can occur anywhere in the text, first need to be removed—a process known as “deidentification” [13]. Even when deidentified, there remains a risk that some identifiable information may have been missed, third parties might be identified, or the narrative may be too revealing.

Routinely collected health data are legally accessed for secondary purposes such as research by 2 lawful bases under UK data protection law: one is the principle of informed consent from the patient and the other is “task in the public interest” [14]. For processing under the lawful basis of “task in the public interest,” health care data needs to be deidentified or anonymized before it can be shared outside the clinical environment under the General Data Protection Regulation principle of data minimization [15]. This is where governance becomes difficult, as deidentifying free-text clinic notes, letters, and reports is complex and a rapidly evolving field, and the accuracy of the process is hard to assess [2]. Technology exists to automatically redact identifying information so that only deidentified documents are presented to computer scientists to develop NLP [1]. However, the reidentification risk from automatically deidentified text remains unknown. Many independent health research ethics committees (RECs) do not have the specialized technical knowledge needed to evaluate the risks posed to patients by this type of research, and indeed, many researchers and data custodians are not sure of the scrutiny and approvals needed to legally process free text for research. As a consequence, a conservative approach is usually taken, resulting in heavy restrictions on data access [16]. Therefore, there are currently very few health care free-text data sets available to NLP researchers to develop and evaluate their algorithms; 1 example is the Medical Information Mart for Intensive Care (MIMIC) database [17,18] in the United States. MIMIC is based on a selected patient population (critical care patients) from 1 US center and contains structured and unstructured (eg, diagnostic reports and physician notes) deidentified data linked to hospital EHR and mortality data. Data that will contribute to the databank remain to be decided and will follow further consultation with stakeholders, but at the very minimum, it will include unstructured text from primary care and hospital records for a defined population of patients in the United Kingdom.

### The Solution

One possibility for breaking down barriers to access to free-text data is to enable access to clinical text via the lawful basis of informed consent. Creating a “donated” databank of clinical free text in which each patient represented has given informed and explicit consent for their data to be used in this way could provide an important and timely opportunity for NLP researchers to develop and train NLP algorithms to process the free text, which can then be used on other data sets in the NHS to conduct clinical research. NLP researchers in universities or other non-NHS settings only need to access a sample of patient free-text data to develop and train the NLP algorithms on the databank, which could then be run on unseen patient free text housed within the NHS for research, avoiding the important privacy issues laid out above.

To test early thinking on the databank, we carried out a series of in-depth focus groups among 4 key stakeholder groups to find out what key stakeholders think about a consented, donated databank of clinical free text to help create, train, and evaluate NLP for clinical research.

## Methods

### Participant Selection and Inclusion Criteria

Four stakeholder groups were identified based on their potential interest and investment in the databank as follows: (1) patients and members of the public, (2) clinicians (NHS general practitioners [GPs], hospital doctors, and doctors in training), (3) NHS Trust information governance (IG) leads and REC members, and (4) NLP researchers based in universities or NHS hospitals. Participants lived in the United Kingdom and were aged ≥18 years. Patients and members of the public were based in the community and had to have some previous knowledge or understanding of the use of free-text health data for research; for example, they may have attended events or workshops on this topic or had experience participating in advisory committees on the use of free text.

### Participant Recruitment

Patients and members of the public were recruited via an advert posted by existing networks, including Health Data Research United Kingdom [19] and the National Institute for Health Research People in Research network [20]. Other stakeholders were identified via existing national networks, contacts, and organizations and were approached directly by email by the research team. In addition, IG leads were identified via the Office of the National Data Guardian [21] and by searching the websites of NHS Trusts and Health Boards. NHS Research Ethics Service committee members were identified by searching the NHS Health Research Authority website [22], and academic NLP researchers were identified via professional networks including the UK health care text analytics network known as Healtex [23].

Potential participants were invited by the research team by email to participate in 1 of the 4 relevant stakeholder focus groups. Before deciding whether to take part, participants were asked to read a study information sheet and return a completed expression of interest form recording basic demographic information including age category (deciles), gender identity, and ethnicity. To understand participants’ views before taking part in the study, they were also asked to indicate how comfortable they might feel about donating their health data for the purposes of the databank outlined in the participant information sheet from 1 of the following categories: very comfortable, somewhat comfortable, not sure, somewhat uncomfortable, or very uncomfortable. Invitees were then sent a consent form by email, which they were asked to complete and sign. Patient and public members were offered a modest financial incentive for participating in the study in line with National Institute for Health Research guidance for recognizing public participation in research [24].

### Focus Groups

Focus groups were conducted on the web on Zoom between March 24 and 31, 2022, and lasted for 90 minutes. A deliberative approach was used where focus groups began with a short presentation on the donated databank by a member of the research team, tailored to each stakeholder group, followed by a question-and-answer session so discussions could be fully informed. The proposed model presented to participants in the prediscussion presentations was of an opt-in approach where people would consent to donate their data to the databank. The facilitator did not direct discussions to confirm whether donated data would be identified or not so that participants could freely share their views around both scenarios.

The team employed a third-party organization with considerable experience in conducting focus groups on the topic of health data to facilitate the groups. Discussions were framed around 4 key questions: (1) Is having a donated free-text databank a good idea? (2) How best could the risks of holding donated, consented potentially identifiable data be managed? (3) What do you think about consent, and how it could be managed? and (4) Who should be allowed access, how should a databank be housed, and for what purposes? Multimedia Appendix 1 presents the questions asked of each stakeholder group.

Discussions were audio recorded, transcribed, and analyzed using thematic analysis.

### Ethics Approval

The study was approved by the University College London REC (0976/002) and complies with the COREQ (Consolidated Criteria for Reporting Qualitative Research) [25] checklist for reporting qualitative studies.

## Results

### Overview

A total of 61 participants took part in the focus groups including patients and members of the public (24 participants), clinicians (10 participants), NHS Trust IG leads and REC members (14 participants), and NLP researchers (13 participants).

In total, 75% (46/61) of the participants recorded their demographic information on their expression of interest form. Of those, 54% (25/46) were female, 52% (24/46) were aged between 31 and 50 years, and 73% (33/45) were White. Overall, most participants (30/46, 66%) were either very comfortable or somewhat comfortable donating their data to the databank, 28% (13/46) were not sure whether they would be willing to donate their data, and only 6% (3/46) of all participants felt either very or somewhat uncomfortable donating their data, all of whom were patients and public members, although the numbers were small (3 patients and public members out of 17; Table 1).

Key findings of the study are summarized in Multimedia Appendix 2.

**Table 1:** Participant demographic information and views around sharing their own data captured before participating in the focus groups.

			All participants (n=46^a^), n (%)		Patients and public members (n=17^a^), n (%)		Clinicians (n=10^a^), n (%)		Information governance leads and research ethics committee members (n=14^a^), n (%)		Natural language processing researchers (n=5^a^), n (%)
**Sex**											
	Female	25 (54)		10 (59)		3 (30)		9 (64)		3 (60)	
	Male	21 (46)		7 (41)		7 (70)		5 (36)		2 (40)	
	Intersex	0 (0)		0 (0)		0 (0)		0 (0)		0 (0)	
	Other	0 (0)		0 (0)		0 (0)		0 (0)		0 (0)	
**Age group (years)**											
	≤30	3 (7)		2 (12)		0 (0)		0 (0)		1 (20)	
	31-50	24 (52)		4 (24)		8 (80)		8 (57)		4 (80)	
	51-65	12 (26)		6 (35)		2 (20)		4 (29)		0 (0)	
	>65	7 (15)		5 (29)		0 (0)		2 (14)		0 (0)	
**Ethnicity**											
	Asian or Asian British	7 (16)		3 (18)		3 (30)		0 (0)		1 (20)	
	Black, Black British, or African	2 (4)		2 (11)		0 (0)		0 (0)		0 (0)	
	Mixed or multiple ethnic groups	1 (2)		1 (6)		0 (0)		0 (0)		0 (0)	
	White	33 (73)		11 (65)		6 (60)		13 (100)		3 (60)	
	Other ethnic group	2 (5)		0 (0)		1 (10)		0 (0)		1 (20)	
**Views around donating data**											
	I would feel very comfortable donating my data	15 (33)		8 (47)		4 (40)		3 (21)		0 (0)	
	I would feel somewhat comfortable donating my data	15 (33)		5 (29)		3 (30)		6 (43)		1 (20)	
	Not sure	13 (28)		1 (6)		3 (30)		5 (36)		4 (80)	
	I would feel somewhat uncomfortable donating my data	2 (4)		2 (12)		0 (0)		0 (0)		0 (0)	
	I would feel very uncomfortable donating my data	1 (2)		1 (6)		0 (0)		0 (0)		0 (0)	

^a^Data are based on 75% (46/61) of the participants who returned this information. Within stakeholder groups, data were returned as follows: patients or public members, 71% (17/24); clinicians, 100% (10/10); information governance leads or research ethics committee members, 100% (14/14); and natural language processing researchers, 38% (5/13).

### Is Having a Donated Free-Text Databank a Good Idea?

#### Perceived Benefits and Challenges

Participants were very enthusiastic about the databank and its intended purpose and saw great value in establishing a platform for development and testing NLP tools to improve their accuracy. Many participants across groups articulated the benefits of producing trustworthy tools to unlock the rich data available in free text, which extended beyond improving NLP methods, including expediting access to, and use of, NHS data by speeding up permissions; accelerating development of NLP tools; and improving health and care leading to better outcomes for patients. The NLP researcher group highlighted its potential value as a training resource to teach and onboard researchers and help familiarize them with free-text data. NLP researchers and clinician groups both welcomed the opportunity to access free-text data for a UK-based population, which would be more appropriate for developing NLP tools on UK health care data sources, moving away from a reliance on US-based data such as MIMIC III or the recently released MIMIC IV [17,18]:

I’d love to see it come to fruition. I think it would be an absolute gold mine.

IG lead and REC member

Don’t let this racehorse designed by [a] committee become a camel, just get something out. I think anything is better than what’s currently offered, which is nothing.

NLP researcher

Several participants in the patient and public group felt that increased access to patient data as a result of the databank may prompt clinicians to improve the quality of their free-text data entry, as they will be more conscious of its wider use:

Very much a great idea. So, the MIMIC dataset I’ve worked with a lot has been really transformative for clinical NLP research in the US. But the MIMIC dataset has some serious issues, in terms of the kinds of data that are included, the representativeness of the sample, and so on, and so forth. So having something that can be created, as a research-specific resource like this, and created with more intentionality, and more design, as to what should be going into it, I think is a really, really incredibly valuable thing to do.

NLP researcher

Despite strong support, participants in all groups advised that clarity around the purpose of the databank; how it will be used; and by whom, both now and in the future, will be essential to its success. Patient and public participants felt this should be made clear in the consent process. Many participants, but patients or public members in particular, expressed concerns around how inaccurate recording, the lack of up-to-date data, or subjective data based on a physician’s own impressions may threaten the aims of the databank, citing frequent instances of errors in their own health records. IG leads and REC members and patients or public participants felt that the accuracy of data must be improved before people trust the outcomes of the databank, and patients or public participants were keen for easier access to their own EHR so they can amend inaccuracies or missing information. However, other participants did not feel data accuracy would be a key factor in the success of the databank, and NLP researchers suggested the databank could provide a unique opportunity for investigating the effect of inaccurate or subjective training data on NLP research findings.

Biases owing to missing data or the lack of generalizability was a considerable concern among all groups. Many felt that donations will be more likely to come from White, middle-class populations and less likely to include people with rare diseases or whose records contain sensitive information. This was seen as most likely to affect data in mental health and social care settings:

There may be intrinsic biases in the actual data that’s getting selected because certain classes of patients, whether it’s by demographics, such as race or income, have more trust in what this is trying to achieve and, therefore, people with less trust won’t actually consent to their data being used and we know, for instance, that that can be quite heavily in race, in the UK, on health data, for instance, and health services and the provision of health services. So, that may introduce biases in the data set.

NLP researcher

I’m not saying it would be necessarily unprofessional, but there could be things that may have been written 10 years ago and that maybe wouldn’t be written now. Is that going to affect your data sets? So, I guess it’s really about the bias that might be there within the unstructured data and whether you’re proliferating that bias by collecting them and then training algorithms.

NLP researcher

Many participants saw artificial intelligence (AI) as playing a key role in health care in the future but were concerned about how AI tools are developed and perform in general. These concerns, which included questions around how tools “decide” which words to analyze, the possibility of scan reports being misread or missing key data, and the accuracy of annotation, are key to feeding into the communication plans for the databank.

#### Data Privacy and Use

Fears around data privacy for both patients and clinicians were raised. Participants discussed complexities in relation to free text, which might act as a barrier to data donation, and how clinicians may be uncomfortable that their identity and views are shared with researchers, for example, where GPs’ personal views around a patient’s health are recorded. Participants advised that such fears might be mitigated to some extent by ensuring robust data security, governance, and transparency around the “data pipeline”—that is, what the databank will be used for and by whom, for example, whether there is any commercial benefit. IG leads and REC members and patients or public participants in particular discussed challenges in articulating how tools may be used in the future and by whom, as technology evolves and society’s views around acceptable and ethical use of their data may change over time. Participants thought that building scenarios for future use into the consent process is therefore important:

My only worry or concerns would be the way that technology develops way into the future and therefore, algorithms, as a result. And it could be ethical now but maybe it would be less ethical in the future.

Patient or public member

#### Types of Data to Include in the Databank and Data Linkages

All participants felt that it is very important that the databank benefit from the inclusion of data from a range of sources to reflect the whole systems approach of the health service and a more integrated care system of the future.

I don’t see how, at this stage, you can start to select what you want to look at because you don’t know what you want to look at. If the purpose of this is to develop algorithms for extracting useful, contextual information then you want the original data, which is being used to train the algorithm, to be as broad as possible.

IG lead and REC member

We are talking about a holistic approach, so that vision should be, I totally agree, whole patient records. And if we are going to use the computers, and train the algorithm, the whole purpose is looking at the wider picture, and bringing it together.

Clinician

Along with primary and secondary care data, some saw value in training NLP tools based on sources that are less commonly used for research, including social services and housing data, to encourage more research in these fields. Perceived advantages of including a broad range of data include introducing a more holistic view of a person’s health needs and including training data based on the different styles of free-text data held in different settings. Some participants discussed the advantages of introducing the databank in phases to ensure timely access and build trust, for example, by starting with 1 health condition such as diabetes or mental health or geographical location.

Such phasing would need to be carefully considered. If condition-specific phasing were to be used, then the imperfections of the diagnostic codes used to select data for a phase would need to be addressed. Solutions might be to use existing NLP applications to identify conditions, accepting that this would have its own limitations, or choosing broader categories of data such as data from specific medical specialties or units:

Our health is based in our experience, and what starts in primary, might end up in secondary. You can’t divide them into two separate things.

Patient or public member

There were mixed views around linking free-text data to other forms of data. NLP researchers particularly valued the opportunity to link the data to other sources, especially mental health data where rich narrative adds important additional detail for research, and some felt that the inclusion of coded data could help verify the accuracy of the free-text data more efficiently. However, some questioned the value of such linkages to train NLP tools and felt linkages, for example, to administrative data, may increase the risk of reidentification and would be resource intensive to manage:

In psychiatry, it tends to be that a lot of the information is locked behind this clinical free text, and they tend to be, by comparison to other medical specialties, a bit more verbose, a bit more narrative, in nature. And so, the ability to incorporate that, and the metadata that’s required to have that, needs to be built in, I think, from the very, very start this has to be extensible, because I’d love to, for example, be able to look at GP notes, and see how they translate over into secondary care, but there are decisions about how this may be structured, early on, that could make that more difficult. So I think that’s something that we need to build in from the start.

NLP researcher

IG leads and REC participants discussed the potential for linkages resulting in “scope drift,” which could lead to the use of data outside of its original purpose. If this occurs, the databank should clearly communicate its wider remit. Scope drift was also a concern for linkages to patient-generated data such as wearable and monitoring devices. Some questioned the accuracy of these data sets and whether this may lead to the development of inaccurate NLP tools, although potential advantages including signaling support for greater use of patient data and “future-proofing” development of NLP tools by including these data, given the inevitable advancement and incorporation of these devices in health care, were also discussed:

This is where, as an IG person, I always start getting nervous and we’re having conversations in our population health because everybody starts saying, “The police, it would be really good if we can put an algorithm together to identify individuals that might start being people that will cause domestic harm.” So, that’s an area that is always a bit nervous to say, “Well, what is that trying to establish,” especially with this project. Because what’s the purpose of having a linked dataset and then trying to do modelling on trying to extract free-text data? I’m not quite sure of the purpose of those two things together.

IG lead and REC member

### Managing Risks of Holding Donated, Consented Identifiable Data

#### Data Privacy and Deidentification

The groups did not discuss a preference for whether data should be left in an identifiable form or should be deidentified; rather, discussions focused broadly on the issue of how to minimize the risk of reidentification, indicating that participants expected data to be deidentified. In particular, the patient or public group highlighted the importance of deidentification alongside data security, including robust data storage and management practices to mitigate risks to data privacy. All participants recognized that eliminating risk completely is unrealistic, but introducing steps to reduce the likelihood of reidentification by using birth year, partial postcode, or a sample of the notes rather than the whole record was discussed. Some clinician participants were conscious of potential legal implications for themselves and supported the removal of clinician details as well. Participants were mindful that the process of deidentification should be carried out on a case-by-case basis. NLP researcher members suggested involving data controllers in agreeing with the approach to deidentification, given their expert knowledge of the data and availability of resources such as data dictionaries to aid the deidentification process. Although rare diseases were regarded as posing a particular risk to reidentification, patients or public participants who themselves have been diagnosed with a rare condition were keen that this should not act as a barrier to the much-needed research and proposed that clear explanations about how data would be used, by whom, and what the data protection issues are might offset concerns. Alongside deidentification, it was felt that following the UK Caldicott Principles, which helps ensure confidential and appropriate use of people’s data [26], adopting strong data security measures to protect against hacks and ensuring that only legitimate, vetted people have access to only the data they need were viewed as key to managing privacy concerns. Patients or public group members in particular voiced the importance of articulating these safeguards to reassure potential donors, and IG leads and REC members suggested that because deidentification is both difficult to define and achieve, there should be an emphasis on defining the purpose of accessing the data and the methods of safeguarding the data:

Is it a pseudonym? Is it a number? Is it aggregated? What level of anonymity is there when we’re discussing this? What’s proposed, or are there different levels (to de-id)?

Patient or public member

If you’re doing a decent level of de-identification so you’re getting masking rates of 90% or something, the risk is going to be very minimal, particularly if you’re providing samples of notes rather than an entire record level. So if you’re looking at medication as your concept for annotation, then you don’t need stuff that doesn’t contain that data, or is unlikely to. So you can start to pick how you pull your sample notes from a record. I think there’s quite a lot you can do to continue to reduce and reduce and reduce that risk, but you won’t eliminate it.

IG lead and REC member

#### Raising Awareness of the Databank

Future consultation with stakeholders, including clinicians, was viewed as essential by all groups, who were keen that engagement be continued to help develop the scope, consent model, and communication plan for the databank. Several participants advised careful planning on how to explain the databank. Patients or public participants expressed the importance of terminology when communicating plans, for example, to clarify what is meant by free text and what is in a health care record that might help allay people’s fears around what they are agreeing to donate:

I want some power. I don’t want to be a passive recipient of this whole data process, which is what’s happened to a lot of us regarding data so far.

Patient or public member

Participants suggested that a targeted, small-scale approach be adopted in the early stages of raising awareness of the databank and starting to gather donations, working with trusted organizations who use health data for research. The involvement of GP practice staff in communicating the databank was seen as important, but clinician participants in particular were skeptical, saying that this was both impractical given their already stretched resources and unnecessary given patients already have the right to agree to their health records being shared. One alternative discussed was to recruit GPs or practice staff who were willing to take on this role. Some ideas for how to reach potential donors included making posters and leaflets available in GP surgeries with a QR code linked to a website about the databank, working with trusted organizations that support the use of health data for research such as *HealthWise* [27] and *use MY data* [28], and identifying community-based “public champions” to advocate the benefits and safety of the databank:

I would share it with patients but I’m constantly limited by time—something else to include in the consultation, so it has to be done through a different delivery mechanism than face to face.

Clinician

### Managing Consent and Offering Choice

Participants expressed the importance of working with trusted NHS and research organizations and providing accessible consent including web-based consent, the availability of audio consent for people with low literacy levels, and translation into multiple languages. The information sheet should clearly set out the intended purpose of the databank and focus on providing reassurance regarding use and data security. If the approach was to include deidentified data, participants wanted this made clear in the supporting materials, possibly aided by including examples of “dummy” data to show what types of data are excluded in the deidentification process. Patients or public members thought that offering people the opportunity to view the databank before consenting might help them understand what information would be included:

People should know what they’re getting involved in. Maybe seeing the database before they opt-in, and also, how far back will the data be taken from...

Patient or public member

Having an actual example of a fake letter or fake clinical notes with a lot of identifiable data and what, actually, will go to the databank in front of you so you see that they did remove all information about you talking about your kids or how your neighbour is annoying you because she is very noisy, all that kind of thing. Maybe, they would see it, that would make a lot of reassurance.

NLP researcher

Patients or public participants felt that offering choice over which data to donate and which type of organization can access the databank might increase donation rates, but others thought this may be complex and resource intensive to achieve and may encourage withholding of sensitive information. The IG leads and REC members group felt that offering choice with a promise to withhold sensitive information is likely to be unachievable and, therefore, undermine trust.

### Who Should Be Allowed Access, How Should a Databank Be Housed, and for What Purposes?

#### Overview

Participants were in favor of access to the databank by both university- and NHS-based NLP researchers. Establishing a “road map” of the types of organizations with which the databank will work was suggested as helpful. Many participants favored a defined approach to access in the early stages of developing the databank, whereby access should be limited to NHS and university-based NLP researchers, and developing a set of standards could ensure that the use of the databank remains in line with its intended purpose. However, the IG leads and REC members group felt that it may be more appropriate to define the types of organizations that can access the databank based on a “compliance” model in which users should show that they can meet a defined level of capability and accountability. Standards should include an assessment of the applicant organization’s motivation for using the databank and their reputation, although the road map should include a well-thought out vetting process to include applicants such as start-ups who may not have a proven track record in trustworthy access to data:

If there were particular requirements around use of the data, commitments to not attempt to re-identify, similar things like that, I think in my view both academic organisations, research students and also research organisations, with the right safeguards, even start-ups, I feel if they can meet a certain level of capability and accountability, then I feel that should be the bar rather than defining the type of organisation.

IG lead and REC member

Participants had mixed views on the use of the databank by commercial organizations including technology and pharmaceutical companies. Patients or public members were generally against the use of their data by these organizations for the purposes of the databank, although other groups felt that these partnerships may be beneficial owing to commercial organizations’ considerable expertise and resources and that they should be allowed access if they could show that they meet the databank’s standards. The primary consideration of commercial access was to ensure adequate return and public benefit. Participants discussed the need for assurances around how tools will be fed back into the public sector, for example, whether the NHS would have discounted access to any tools that were developed as a result of the databank. Access by charities was viewed as potentially problematic, as charities are less regulated and often have a campaign focus, which may result in data use being less scrutinized or controlled and the creation of NLP tools biased toward certain outcomes. Government organizations, insurance companies, and lawyers were deemed unsuitable.

Several participants agreed that existing models of good practice about access such as the Secure Anonymised Information Linkage (SAIL) Databank [29] in Wales should be incorporated and that learning from the Centre for Data Ethics and Innovation Public Attitudes Survey on Data and AI [30] about who is trusted with data and in what circumstances should inform the road map.

#### Fee-Based Model for Use of the Databank

Clinician and NLP researcher groups were asked about how the databank should be funded. Both groups welcomed government funding to help develop the databank, but a fee-based model was viewed as more sustainable in terms of supporting the management of access and oversight, ensuring data quality, and allowing data to be updated over time. Charging a not-for-profit fee was viewed as realistic, and participants favored the development of a tiered costing model with different levels of access and cost depending on the user, their reason for access, and the volume of data requested. Suggestions for tiered access included providing an institute-wide membership fee to enable access for anyone working within the organization and discounted rates or free access for those who contribute to data donation, maintenance of the databank, or data cleaning or other data quality control. It was suggested that the development of the cost model should be informed by existing approaches such as the Linguistic Data Consortium [31], which supports NLP research by creating and sharing resources, and the UK Data Service [32], a large repository of economic, social, and health data sets for research and teaching.

#### Data Gathering, Management, and Housing

Participants felt that with health care services under notable pressure, a robust and sustainable costing plan to support the transfer of data into the databank is essential. For example, GP practices may expect to be paid to carry out data extraction for the databank; therefore, costs should be factored into the costing model.

Services are already stretched and I know in some of my practices if you come to them and ask them for data, whether patient’s consented or not, they’re going to tell you to go take a high jump. I’m not going to spend my time extracting that for you or printing that off and sending it to you because I haven’t got the time or capacity.

Clinician

The participants articulated 5 considerations that should be built into the way the databank is managed. The considerations were that the databank should be (1) accessible: access should be easier than the current process of applying for and accessing data from data providers; (2) up-to-date: the databank should be supplemented with new data so NLP tools are trained on current information; (3) controlled: robust technical controls should be put in place as the primary mechanism for managing data, including allowing and revoking access; (4) tracked: a mechanism should be in place to allow the use of data to be tracked in real time to see what happens to the data after they are accessed; and (5) transparent: transparency around how the databank is being used to track public benefit, for example, publishing details on a website, was seen as essential.

There was no clear consensus on the best approach to housing the databank. The options discussed included housing it within the NHS (perceived benefits were trustworthiness and existing robust policies around data breaches or data misuse; disadvantages were possible lack of technical infrastructure or resources to be able to manage it effectively) or a university setting. Different models considered included adopting a partnership approach in which the databank could be housed within a university but be governed by the NHS. Storing data within a secure environment such as a trusted research environment where data cannot be removed was felt to be appropriate, and existing models of good practice should be drawn upon, for example, Genomics England [33] and Health Data Research United Kingdom [19].

#### Oversight and Management

The integrity of the databank was closely linked to the approach to oversight and clear communication of how gatekeeping will take place. Most participants favored the establishment of an oversight committee to consider the range and types of data collected, review applications for access and use, ensure transparency around use including what the NLP tools being developed will be used for, and monitor and review data safeguarding. The committee should be independent and consist of people with diverse demographics, backgrounds, and expertise, including experts in the use of data, data controllers, and lay representatives. Patients or public participants discussed the importance of ensuring that the application process to join the oversight committee was accessible and did not put off potential new applicants by overemphasizing a requirement for previous experience, as is often the case.

## Discussion

### Principal Findings

This study set out to test early stakeholder thinking around the acceptability and design considerations for the creation of a consented donated databank of clinical free text to develop and test NLP methods and tools. Understanding the details to inform establishment of such a databank was highlighted as a key recommendation in a recent position paper on the development of data governance standards for using clinical free-text data in health research [1]. All stakeholder groups voiced strong support and a pressing need for a free-text databank for the purposes set out to them. Participants highlighted a range of complex issues for consideration as the databank is developed, but there was a plea, particularly among the NLP researcher group, to move with haste to design something that works without becoming overburdened with the many complexities. One suggested approach was to develop the databank in phases, with the initial phase focusing on a specific health condition or type of health data, to test out whether people are willing to donate their data for this purpose and how it would work. Although not raised by any of the groups, a sensible starting point for the databank may be to exploit existing cohorts such as Generation Scotland or the UK Biobank where participants have already consented to share their data for research and can be easily contacted to invite them to donate their data to the databank. Participants stressed the importance of ongoing engagement and involvement with stakeholder communities in the development and operation of the databank. This position encapsulates the widely held view of the importance of transparency to increase the general lack of awareness about how patient data are used, by whom, and for what purposes [16].

Future proofing the databank at an early stage was viewed as important to take into account how uses of the databank and advances in technology might change over time. Participants also highlighted the importance of ensuring that plans for the provision and maintenance of the databank are sustainable in the future. There was a general agreement among stakeholder groups that the databank should draw upon a range of data sources to ensure that NLP tools reflect an integrated care system in the future, although there were mixed views about the benefits of linking to other data sources. The types of data to be included in the databank (eg, structured data in the GP or hospital record that may improve the performance of existing models owing to the inclusion of text-based features [34,35] or linkage to other data outside the NHS EHR, eg, national registry, mortality, or administrative data) should reflect stakeholder views on acceptability and practicalities, including cost, especially at the start. These issues should be explored in more detail in the next phase of the study. Participants agreed that the way the databank is created will be crucial to its success. The importance of communicating the intended purpose and approach to accessing the databank and the proposed mechanisms for safeguarding the data came up at numerous points during discussions with all groups. Learning from existing examples of good practice around access to data, data security, and how to fund the databank was also deemed crucial.

During the discussions, participants were not directed by the facilitator as to whether data stored in the databank would remain identifiable or not to encourage a broad conversation around anonymity in relation to the databank. Although it was made clear that donations would be based on explicit donor consent, issues of trust and maintaining patient confidentiality featured strongly in the discussions, particularly among the patient and public and IG and REC groups who were aware that public awareness of the risk of reidentification might act as a barrier to data donation, particularly among people with rare diseases who may be easier to identify. Interestingly, only patients and public participants expressed concern about sharing their own data with a databank (Table 1), although the numbers were small, with only 18% (3/17) of the patients or public members declaring that they would be very or somewhat uncomfortable donating their data. This finding is likely to reflect both a clearer understanding of the potential benefits of the databank among other stakeholder groups, given their expertise in this area, and the complexity of what is required to manage and mitigate the risks of data breaches and uphold privacy, which other stakeholder groups may understand more clearly. More work is needed to engage with patients and public members in this area to develop strategies for clear and widespread articulation of the benefits of the donated databank. Given the nature of the data and the challenges of removing personal identifiers, a realistic approach to deidentification will need to be adopted and made transparent and should be supported by robust processes to protect against risks. The deidentification approach may need to be dynamic, depending on the type of data and health condition, as some identifying data might be essential to the research study, for example, if the databank will be used to develop and test deidentification tools. It will also be worth exploring options to replace identifiers with random replacement identifiers to enable this type of work and remain mindful of the advances in generating synthetic data. Further exploration of stakeholder views to understand if stakeholders expect data to be stored in an identifiable form or if they want it to be deidentified is warranted. Although stringent steps will be adopted to minimize the risk of reidentification of patients by deidentifying the data and ensuring strict controls over who can access the data and how data will be made available, for example, within a trusted research environment, risk cannot be eliminated completely. The model for the databank should balance strong governance and security measures to ensure that access is not unnecessarily burdensome or complex. Learning from existing models will be the focus of the next phase of the development of the databank.

Another clear theme throughout the discussions was the need to develop a carefully planned and strong communication plan to build trust. It was suggested that distinct key messages be prepared depending on the stakeholders’ interests in the databank. For example, there should be targeted communication with clinicians regarding their data privacy and with data controllers regarding data security. Communication should incorporate relevant background information (eg, to counter the lack of awareness of what data are contained within EHRs) and address the context for the databank, which drives donations and the involvement of GP practices and other data providers.

Ideally, linguistic features in NLP should be representative of the entire population to ensure that the findings are not biased and are representative across patient groups. However, it is also important that the databank reflects what happens in the real world, despite the potential limitations owing to bias. The participants discussed the potential for bias and its impact on the databank (“garbage in, garbage out”) in 2 areas: first, potential biases in the data because of inaccuracies or missing data, and second, potential biases in the data because of donations that lacked demographic variation. Biases in the annotation process [36] and other potential biases have not been discussed. Further in-depth consideration of how to avoid biases and the potential consequences for the trained models is needed when developing the databank and should be addressed clearly in the communication plan.

Participants expressed mixed views about the impact of training NLP models on inaccurate or missing data or data that are not up-to-date. Patients or public participants were concerned that the quality of data would affect the quality of the algorithms that will be developed. Patients or public participants were able to highlight numerous examples of inaccurate or missing data in their own health records which they found concerning and they expressed concern around how the potential lack of accurate or up-to-date data might impact on trust in the databank. Additional engagement with stakeholders, in particular patients and public participants, should be carried out to tease out and address major questions or lack of understanding about the impact of accuracy, subjectivity, and representativeness of data when training NLP tools so that people have a better understanding of what the databank can achieve. Communication should include efforts to make clear that NLP development is not interested in whether data are accurate or true, as it is simply trying to learn the linguistic properties of the data and how they relate to target concepts defined by NLP researchers and annotators.

Notably, participants were acutely aware of, and made reference throughout the discussions to, the important role AI will play in health care in the future but raised concerns about how AI tools are developed and perform and what might be ethical in the future. Embedding ethical approaches in developing data-driven technologies for AI and understanding public trust is high on the UK governments’, researchers’, and other stakeholders’ agendas. For example, the NHS England Accelerated Access Collaborative [37] is committed to working with patients to ensure that AI innovations reflect the priorities of the end users and support innovators to embed public involvement in the development of AI technologies. The Centre for Data Ethics and Innovation, which is responsible for monitoring public attitudes toward data and AI over time, recently published findings from its second “Public Attitudes to Data and AI (PADAI) Tracker Survey” [30]. Findings from our study reflect similar views to those identified in this survey: for example, data security and privacy remain major concerns, people expect strong governance overseen by experts, and trust is strongly linked to the level of trust in the organizations that are accessing the data. Adhering to best practices around ethical AI principles and frameworks and anchoring public involvement in the development of the databank should be a priority to build trust, and developers of the databank should engage with leaders in the field to ensure this is embedded in plans for the databank, for example, the NHS England AI Ethics Initiative [38]. To keep up with the research and development in AI applied to clinical settings that is happening in the United States (made possible by data sets such as MIMIC III and IV), the UK government should channel resources into funding such a databank to harness rapid advances of AI technology and support long-term investment in the AI ecosystem in the future.

### Limitations

This study has limitations. The sample size was relatively small, and there was a lack of diversity, particularly from younger and older participants and people of color. Therefore, the participants’ views are unlikely to be representative of the UK general population. Engagement with more diverse groups and stakeholders who were not included in this work, for example, data controllers, is essential when planning next steps for the databank in the future. Thematic analysis does not allow views to be quantified, so we were unable to report how many participants felt a particular way. Furthermore, the aim of the focus groups was not to produce a specific set of recommendations. Rather, our findings provide useful insights into initial thinking, and the recommendations presented in this study (Textbox 1) therefore reflect a set of potential suggestions and advice based on the views of participants generally. Web-based discussions were limited to 90 minutes to ensure that the length of the focus groups was manageable for participants, which meant some topics could not be explored in depth. The research team therefore agreed in advance how to limit questions to ensure topics deemed the most relevant to particular stakeholders were covered within the time frame. Opportunities to explore topics in more detail will be sought in the next phase of the study.

**Proposed recommendations and suggestions for setting up the databank.**

**General approach**

- Recommendation 1: Stakeholders should be involved throughout the development, implementation, and maintenance of the databank, including development of the scope, consent model, and communication plan.
- Recommendation 2: The databank should draw on the existing successful examples that can offer helpful models for consent, governance, data housing, and data security and be governed by an oversight committee.

**Scope and phasing of the databank**

- Recommendation 3: The databank should have a clearly defined purpose and take into account how natural language processing (NLP) researchers may wish to use it in the future.
- Recommendation 4: Development of the databank should be based on a small-scale, gradual approach to starting to gather donations to establish proof of concept and interest in donating to the databank. This might involve gathering data for 1 health condition (eg, diabetes) or location (eg, a mental health National Health Service [NHS] Trust) before moving to include others.

**Channels to reach potential donors**

- Recommendation 5: Reaching potential donors and publicizing the databank should include trusted individuals, networks, and organizations that support research using health data.
- Recommendation 6: Innovative ways to reach out to minority groups such as identifying public community champions who can advise and reassure others about the benefits and safety of the databank should be explored.

**Consent**

- Recommendation 7: Ensure the consent process is simple and accessible. Consent should be collected electronically, and information should link to a relevant NHS research ethics committee website and be offered in multiple formats and languages.
- Recommendation 8: The focus of the consent information sheet should be to provide reassurance around use; data security; and, if appropriate, deidentification. It should clearly define the purpose of the databank, provide a clear explanation of what data people are being asked to donate, and describe examples of scenarios for future use.
- Recommendation 9: Opportunities for showing potential participants their own personal health record before consenting should be explored.

**Communication**

- Recommendation 10: A clear and comprehensive communication plan should be carefully planned and developed with targeted messages for the different stakeholders (eg, clinicians regarding their data privacy and data controllers regarding data security).
- Recommendation 11: Communication should cover the following key elements clearly to build trust in the databank: predonation involvement (eg, possibility for participants to see their personal data and amend errors before donating); general aspects around data (what is free text? and what data are in a health care record?); content (what data are to be donated?); purpose (what the free-text data will be used for and by whom?); different contexts that NLP tools will be used in (eg, will data be used largely for commercial benefit?); and, crucially, the public benefit that NLP tools trained on the data could bring.

**Pathways to databank access**

- Recommendation 12: The foremost consideration for access should be to ensure public benefit and that benefits of data use are shared equitably.
- Recommendation 13: A “road map” should be developed to include the types of organizations the databank will work with, based on a compliance model where users should show they can meet a defined level of capability and accountability. The road map should include a set of standards and approach to “due diligence” to ensure databank use is in line with its intended purpose. Access could be granted based on an organization’s ability to meet, and commitment to comply with, the standards and an assessment of the applicant organization’s reputation and motivation for using the databank, rather than limiting which types of organization should be allowed access. The road map should incorporate recent learning on who is trusted with data and in what circumstances.
- Recommendation 14: Although development of the databank is in its infancy, it may be prudent to limit access to a small group such as NLP researchers linked to the NHS and UK universities.
- Recommendation 15: Access should be easier than the current process of applying for and accessing data from data providers.

**Cost model for the databank**

- Recommendation 16: A clear, transparent, and not-for-profit fee-based model should be developed that ensures sustainability of the databank over time. Fees should be used to maintain the database, support and manage access and standards, support oversight, ensure quality of data, and support updating the databank with new data over time.
- Recommendation 17: A tiered access model should include different levels of fees depending on the user, reason for use, and volume of data required (eg, access to a portion of the data set or all of it). Discounted or free access should be considered, for example, discounted access for organizations that contribute to data donation or where the databank would be used for teaching purposes. “In kind” arrangements could be considered for organizations that collaborate on improving quality of data (eg, cleaning data for access).
- Recommendation 18: Data provider costs to extract the data for the databank should be factored into the model.

**Range and types of data to include in the databank**

- Recommendation 19: A range of data types across different settings should be included in the databank.
- Recommendation 20: The databank should be kept up-to-date so that NLP tools are trained on current information.

**Governance and oversight**

- Recommendation 21: An independent oversight committee that has no stake in the NLP tools being developed should be established to monitor and review applications for data use and ensure transparency around use (eg, what the NLP tools being developed will be used for, the range and types of data included in the databank, who is using the databank, and safeguards to protect the data).
- Recommendation 22: The oversight committee should include members from a range of diverse ethnic and sociodemographic backgrounds and expertise, including data experts, data controllers, and lay representatives. Experts in specific data domains may be brought into the committee on an ad hoc basis to advise on specific applications.
- Recommendation 23: Ensure the process for applying to join the oversight committee is accessible and does overemphasize the need for previous experience on such committees.
- Recommendation 24: Lay members should be paid for their time serving on the committee.

**Databank housing, management, and security**

- Recommendation 25: The databank should be housed in a university or NHS trusted research environment.
- Recommendation 26: Robust technical controls should be put in place as the primary mechanism for managing data, including allowing and revoking access.
- Recommendation 27: A mechanism should be put in place to allow the use of data to be tracked in real time to see what happens to the data after they are accessed.
- Recommendation 28: Transparency around how the databank is being used to track public benefit should be ensured, for example, publishing details on a website.

**Approaches to maintain participant anonymity**

- Recommendation 29: Further engagement with stakeholders should be carried out to explore views around whether data should be stored in an identifiable or deidentified form in the databank and expectations around deidentification.

### Comparison With Prior Work

Although the focus of this study centered on creation of the databank that has not, to the authors’ knowledge, been previously explored, there were several areas where themes overlapped with previous research on attitudes toward the use of free-text data for research, which has been discussed particularly among patients and the public [16,39]. Although several benefits highlighted by participants in this study related specifically to the databank, wider benefits discussed included the potential for improving health and care leading to better outcomes for patients, which mirrored benefits identified by other UK research studies that used clinical free text [2]. Despite acknowledging a broad range of potential benefits, participants raised a number of concerns, particularly around how AI tools are developed and perform in general, the effect of possible biases, privacy risks, and reidentification. Previous research on potential harms of the use of free-text data for research has shown that the public harbors similar concerns around the use of free-text data for research generally, despite no evidence of these harms actually taking place following data breaches [40]. The issue of trust was raised several times, as was the importance of clear communication and a transparent approach to help build trust. Participants in this study felt that trust is strongly linked to the level of trust in the organizations that access the data, which echoes findings from other studies that showed that the public evaluates the trustworthiness of research organizations by assessing their competence in data handling and motivation for accessing the data [41]. A Citizens’ Jury on the use of free text for research carried out in 2018 [16] found a high degree of willingness to share EHR data for public benefit among public participants who were informed about the use of free-text data, although participants expressed caution owing to concerns around the lack of transparency in the use of data and increased privacy risks. Participants in the Citizens’ Jury suggested keeping patients informed about the use of their data and being transparent about ways to opt out of data sharing. These attitudes were mirrored in this study, as were views on risks related to deidentification of free text, which were in line with previous findings, including concerns around accuracy of removing patient identifiers.

### Next Steps

The recommendations and advice resulting from the study are summarized in Textbox 1. The findings will be used to plan the next phase of developing the databank, including a pilot study to design the road map and communication plan and test the feasibility of donating to the databank. Next steps will involve identifying and reaching out to a broad range of stakeholders based on their diverse knowledge and skill sets to develop the vision for the databank and inform the road map and standards, including researchers, patients and public members, governance experts, providers of NHS, data controllers, charities, government, and industry. The road map and standards could be further informed by a national web-based survey that will be co-designed with stakeholders to explore in more detail the acceptability and design considerations highlighted in this study, including understanding whether stakeholders expect data to be stored in an identifiable or deidentifiable form. Planning the next steps will draw on recommendations across relevant themes that were highlighted in a position paper on developing data governance standards for the use of free-text data in health research, including the involvement of patients and public members at identifiable data stages and opt-in consent models for the reuse of free-text data [1].

### Conclusions

Improved access to clinical free-text data will help support technological innovation for developing novel and valid NLP tools to support research for public benefit. One way to leverage access is through the creation of a consented databank to develop and train NLP tools outside the NHS via the lawful basis of informed consent. This study showed strong multistakeholder support for a databank for this purpose and an urgent need to move forward to develop something quickly. Stakeholders expressed commonality around many issues such as governance, communication, and sustainability, but there were also stakeholder-specific concerns such as clinician concern around increased workload and privacy and patient-and-public concern around inaccuracies in their personal EHRs and how their data will be used. These issues should be explored in more detail and targeted among individual stakeholder groups. Findings from this study will be used to inform the next steps for adopting a partnership approach to establish a national, funded databank of free text for use by the research community.

## Appendix

Multimedia Appendix 1 Topic guide: focus group questions and the distribution of questions between groups.

Multimedia Appendix 2 Summary of focus group findings and key areas of discussion.

## Abbreviations

AI: artificial intelligence
COREQ: Consolidated Criteria for Reporting Qualitative Research
EHR: electronic health record
GP: general practitioner
ICD-10: International Classification of Diseases, 10th revision
IG: information governance
MIMIC: Medical Information Mart for Intensive Care
NHS: National Health Service
NLP: natural language processing
REC: research ethics committee
SAIL: Secure Anonymised Information Linkage
SNOMED CT: Systematized Nomenclature of Medicine Clinical Terms

## Data Availability

Focus group discussions were recorded for the purposes of report writing. Recordings were destroyed after being transcribed, but transcriptions are stored securely for 24 months, after which they will be destroyed (May 2024).
