# Supplementary material for: Understanding Views Around the Creation of a Consented, Donated Databank of Clinical Free Text to Develop and Train Natural Language Processing Models for Research: Focus Group Interviews With Stakeholders
Source: JMIR Med Inform. 2023 May 3;11:e45534. doi: 10.2196/45534 (PMC10193205; doi:10.2196/45534)
Supplement: Multimedia Appendix 1 [file medinform_v11i1e45534_app1.doc]

**Appendix 1. Topic guide: Focus group questions and distribution of questions between groups**

| **Questions** | **Patient and public group** | **Clinician group** | **Natural Language Processing Researcher group** | **Information Governance /Research Ethics Committee group** |
| --- | --- | --- | --- | --- |
| 1. **General attitudes towards the creation of a free text databank** | | | | |
| Is having a donated free text databank to develop methods for health research a good idea? | **✓** | **✓** | **✓** | **✓** |
| What are the ethical implications of sharing free text data for the purposes outlined here that may not be applicable to sharing coded data? |  |  |  | **✓** |
| 1. **Attitudes around which data are acceptable for donation to the databank** | | | | |
| Which data (primary or secondary care or both) do you think should be included in the databank? | **✓** | **✓** | **✓** | **✓** |
| Do you feel differently about inclusion of any of the following types of data (primary or secondary care/other)? If yes, why/what are your concerns? | **✓** | **✓** | **✓** | **✓** |
| How much control should a patient have over what is included and what is not included in the databank? |  | **✓** | **✓** | **✓** |
| What do you think about the inclusion of some other patient-specific structured data that helps researchers to make sense of the data, like demographics, setting, or diagnosis codes and other information or linkage to other data that would help make sure the data is correct and useful? |  | **✓** | **✓** | **✓** |
| 1. **Attitudes towards use and access of a donated free text databank** | | | | |
| What should the databank be used for? | **✓** | **✓** | **✓** | **✓** |
| What should it not be used for? | **✓** | **✓** | **✓** |  |
| We anticipate that UK researchers (clinical and non-clinical) based in NHS  hospitals and universities will require access to the data in the databank. How do you feel about making the databank accessible to other groups of people for the intended purpose of developing methods for health research? Which groups? Why? | **✓** | **✓** |  | **✓** |
| Who should not get access? Why? | **✓** | **✓** |  |  |
| Should access be free or should users pay a fee towards management of  the databank? |  | **✓** | **✓** |  |
| Where should the database be housed? |  | **✓** | **✓** | **✓** |
| How should access and use of the databank be managed? |  | **✓** | **✓** | **✓** |
| 1. **Attitudes towards managing risk of a donated free text databank** | | | | |
| What level of anonymisation of the data would you be comfortable with? (e.g. only remove names or remove additional details such as date of birth, address, other)? / What level of anonymisation of the data should there be? | **✓** | **✓** | **✓** | **✓** |
| Any other ways of managing risk that would be important to you? | **✓** | **✓** | **✓** |  |
| 1. **Attitudes towards managing participation and consent for donating personal free text to the databank** | | | | |
| How would you like to be approached or informed about the opportunity to donate your free text data to the databank? / How should patients be approached or informed about the opportunity to donate their free text data to the databank? | **✓** | **✓** |  | **✓** |
| How would you feel about displaying posters for patients on the databank in your clinic? |  | **✓** |  |  |
| If a patient gives their consent to donate their data to the databank and you receive a request to share their data, how would you like this process to be managed? |  | **✓** |  |  |
| How would you want to be consented to the databank? / How should patients be consented to the databank? | **✓** |  |  | **✓** |
| 1. **Benefits and value of the databank to patients** | | | | |
| How should we articulate benefits and opportunities of the databank in a way that is accessible and meaningful to patients and the public? | **✓** |  |  |  |
| How do patients and the public want to be included in decision-making panels and wider oversight of the databank? | **✓** |  |  |  |
