# Supplementary material for: Understanding Views Around the Creation of a Consented, Donated Databank of Clinical Free Text to Develop and Train Natural Language Processing Models for Research: Focus Group Interviews With Stakeholders
Source: JMIR Med Inform. 2023 May 3;11:e45534. doi: 10.2196/45534 (PMC10193205; doi:10.2196/45534)
Supplement: Multimedia Appendix 2 [file medinform_v11i1e45534_app2.docx]

**Appendix 2.** Summary of focus group findings and key areas of discussion.

| Topic/theme | | Summary of key points raised during focus group discussions |
| --- | --- | --- |
| **Is having a donated free-text databank a good idea?** | | |
|  | Perceived benefits | - Accelerating development of, and producing, trustworthy NLP^a^ tools - Improving NLP methods - Move away from reliance on US data sets to allow access to United Kingdom–based free-text data, which are more appropriate and generalizable for research using UK health data - Expediting access to, and use of, NHS^b^ data for research - Facilitating research using free text will lead to better outcomes for patients - Use as a training resource to teach and onboard researchers - May prompt clinicians to improve the quality of their free-text data entry - May provide an opportunity for investigating the effect of inaccurate or subjective training data on NLP research findings |
|  | Perceived challenges | - Potential bias owing to missing data or lack of generalizability - Inaccurate recording, lack of up-to-date data, or subjective data based on physician’s own impressions may threaten the aims of the databank - Lack of accurate data may impact on trust in the outcomes of the databank - Providing patients with easier access to their own health records so that they can amend inaccuracies or missing information - Concerns around how AI^c^ tools are developed and perform |
|  | Data privacy and use | - Complexities around free text and possibilities of reidentification might act as a barrier to data donation - Clinician discomfort around identification and sharing of their personal views with researchers - Challenges articulating how tools may be used in the future and by whom |
|  | Types of data to include in the databank | - Include data from a range of sources to reflect the whole systems approach of the health service and the integrated care system of the future - Training NLP tools based on data sources that are less commonly used for research including social services and housing data may encourage more research and introduce a more holistic view of a person’s health needs. - The databank might benefit from being introduced in phases to ensure timely access and build trust, for example, starting with 1 health condition or 1 geographical location. |
|  | Linkage to other data | - There were mixed views about the value of linking free-text data in the databank to other forms of data. - Linkage to mental health data, which contains a rich narrative, could add important additional detail for research, or coded data may help validate the accuracy of the free-text data. - Linkages may be resource intensive to manage or increase the risk of reidentification (eg, linkage to administrative data). - Linkages could result in “scope drift,” leading to the increased risk of data being used outside of its original purpose. - Linkages to data from wearable devices may lead to development of inaccurate NLP tools if data are not accurate, although advantages included signaling support for greater use of patient data and “future-proofing” development of NLP tools, given the increasing use of such devices in health care. |
| **How best could the risks of holding donated, consented, and identifiable data be managed?** | | |
|  | Data privacy and deidentification | - Eliminating risk completely is unrealistic and should not impede the much-needed research. - Clear explanations about how data would be used, by whom, and the methods of safeguarding the data might offset concerns for potential donors, and there should be an emphasis on defining the purpose of accessing the data. - Deidentification should be considered alongside the measures of robust data security including data storage and management practices to mitigate risks to data privacy. - Data controllers should be involved in agreeing the approach as to how data should be deidentified, given their expert knowledge of the data and availability of resources to aid the deidentification process. |
|  | Raising awareness of the databank | - There should be ongoing consultation with stakeholders to help develop the scope, consent model, and communication plan for the databank. - A multistakeholder approach is important to raise awareness and build trust among groups who have influence over donations, such as clinicians or data controllers. - Providing clear explanations of key terms and concepts is important when communicating plans for the databank, and it will increase trust. - Involvement of GP^d^ practice staff in communicating the databank may not be practical, and staff may need to be recruited to take on this role. - Raising awareness could include making posters and leaflets available in GP surgery waiting areas with a QR code linked to a website about the databank, partnering with trusted organizations who support use of health data for research and could highlight the databank in their newsletters, and recruiting public “champions” to advocate the benefits and safety of the databank. |
| **What do you think about consent, and how could it be managed?** | | |
|  | Channels for consent | - Practical and accessible consent processes should be considered. - Clinician involvement in the consent process is likely to be impractical. |
|  | Information to support consent | - Materials should focus on setting out the intended purpose of the databank and providing reassurance around use and data security (and, if appropriate, deidentification of data) to increase donor confidence to donate all their data. - Offering people the opportunity to view the databank before consenting might help people understand what information in included. - Providing examples of “dummy” data that are excluded in the deidentification process might aid potential donors’ understanding of the process. |
|  | Patient control over what data they donate and who will have access | - There were mixed views around the benefits of offering choice over which data to donate and which type of organization can access the databank. - Participant choice over what data to donate may result in increased donation rates. - Participant control over which data to donate may be too complex and resource intensive to achieve. |
| **Who should be allowed access, how should a databank be housed, and for what purposes?** | | |
|  | Developing a framework around access and use of the databank | - A set of standards should be developed to ensure that use of the databank remains in line with its intended purpose and set out how to assess the applicant organization’s motivation for using the databank and their reputation. - A “road map” should be developed, detailing the types of organizations the databank will work with and setting out the conditions under which organizations can access the data. - Start small: develop a defined approach limiting access to NHS and university-based researchers in the early stages of developing the databank. - Explore and integrate existing models of good practice around access to data (eg, the Secure Anonymised Information Linkage(SAIL) Databank) [29] so as not to “reinvent the wheel.” |
|  | Who should have access? | - The primary consideration around access should be around ensuring adequate return and public benefit. - Organizations must provide assurances around how tools will be fed back into the public sector. - NHS and university-based researchers were deemed acceptable users of the databank. - Mixed views around the acceptability of commercial organizations that could be considered suitable if they can show that they meet the databank’s standards - Charities are less regulated, and their motives should be scrutinized to avoid creation of NLP tools biased toward certain outcomes. |
|  | Fee-based model for use of the databank | - The databank should operate as not-for-profit. - GP practices and hospitals are overstretched, and the model should support payment for them to support carrying out data extraction for the databank. - A tiered, fee-based model would support provision, management, and oversight of the databank into the future. |
|  | Data gathering, management, and housing | - A robust and sustainable plan for how data will be transferred into the databank is essential. - Access should be simpler and quicker than the current process of applying for and accessing data from data providers. - Data should be refreshed with new data extracts so that NLP tools are trained on current information. - Robust technical controls should be enabled to manage data including allowing and revoking access. - Processes should be put into place to allow use of data to be tracked in real time to see what happens to the data after they are downloaded. - There should be transparency around how the databank is being used so as to track public benefit. - Data should be securely stored within a trusted environment, drawing on the existing models of good practice. - Housing the databank within the NHS, which was seen as trustworthy, but it may lack the technical infrastructure or resources to be able to manage it effectively. - A partnership approach where the databank is housed within a university but governed by the NHS should be explored. |
|  | Oversight and management | - Integrity of the databank was viewed as closely linked with the approach to oversight and clear communication of how gatekeeping will take place. - An independent oversight committee should oversee the key aspects of the databank and monitor and review data safeguarding. Members should include experts in the use of data, data controllers, and lay representatives with diverse demographics, backgrounds, and expertise. - The application process to appoint members to the oversight committee should be open and accessible. |

^a^NLP: natural language processing.

^b^NHS: National Health Service.

^c^AI: artificial intelligence.

^d^GP: general practitioner.
